# Efficacy of Interventions to Prevent Physical and Sexual Dating Violence Among Adolescents: A Systematic Review and Meta-analysis

**DOI:** 10.1001/jamapediatrics.2021.4829

**Published:** 2021-11-29

**Authors:** Antonio Piolanti, Heather M. Foran

**Affiliations:** 1 Health Psychology Unit, Institute of Psychology, Universität Klagenfurt, Klagenfurt, Austria

## Abstract

**Importance:**

Sexual and physical dating violence is prevalent among adolescents and is associated with adverse health effects.

**Objective:**

To conduct a systematic review and meta-analysis of randomized clinical trials to assess the efficacy of prevention programs for sexual and physical dating violence in adolescents.

**Data Sources:**

Search terms were combined for dating violence, adolescents, and randomized clinical trials in PsycINFO/Eric/PsycArticles, PubMed, and Web of Science databases from inception through April 2021.

**Study Selection:**

Included studies had a randomized design of any type examining the efficacy of an intervention to reduce dating violence among adolescents and provided at least 1 measure of sexual or physical dating violence.

**Data Extraction and Synthesis:**

Data extraction coded characteristics of trials, participants, and interventions and assessed risk of bias using the Revised Cochrane Collaboration Risk of Bias tool. Outcomes were pooled using a random-effects model. Subgroup and meta-regression analyses were performed to explore the target population and intervention factors associated with positive outcomes.

**Main Outcomes and Measures:**

The odds ratio (OR) was calculated for 3 different outcomes: (1) sexual dating violence, (2) physical dating violence, and (3) composite measures of sexual and physical dating violence. For each outcome, separate analyses were conducted for survivorship and perpetration scores. We also combined the scores of physical/sexual violence and perpetration/survivorship of teen dating violence into a single composite overall outcome including all studies.

**Results:**

Eighteen trials (22 781 adolescents) were included. Overall, interventions were associated with reduced physical and sexual dating violence (OR, 0.78; 95% CI, 0.69-0.89; *P* < .001). Separate analyses further indicated that this association was significant for physical violence perpetration (OR, 0.74; 95% CI, 0.59-0.92; *P* = .01) and survivorship (OR, 0.78; 95% CI, 0.64-0.95; *P* = .01). For sexual violence, the association was not statistically significant. Exploratory subgroup analyses revealed that trials targeting at-risk youth, older adolescents (age >15 years), and trials involving parents in the intervention reported significantly larger effect sizes. Meta-regression analyses did not show any significant associations between intervention effect sizes and length or intensity of the programs. Publication bias was observed, but the adjusted ORs remained significant. Three studies reported iatrogenic associations.

**Conclusions and Relevance:**

Findings from this study suggest that prevention programs may be effective in reducing physical teen dating violence, but there is unclear evidence of the effect on sexual violence outcomes. Further research assessing the active ingredients of interventions, especially in the area of sexual dating violence, is warranted.

## Key Points

**Question:**

What is the efficacy of prevention programs for physical and sexual teen dating violence?

**Findings:**

In this systematic review and meta-analysis of 18 trials including 22 781 adolescents, the implementation of interventions targeting dating violence among adolescents was associated with a significant reduction in overall physical and sexual violence. However, when examined as separate outcomes, a significant reduction was found for physical violence only.

**Meaning:**

Prevention programs may be effective in reducing physical dating violence among adolescents; unclear evidence on sexual violence outcomes highlights the need for further research studies.

## Teaser

This systematic review and meta-analysis evaluates the efficacy of interventions in reducing physical and sexual violence in adolescents’ dating relationships.

## Introduction

Physical and sexual teen dating violence (TDV) is common among adolescents. Research studies have reported a prevalence of approximately 20% for physical and 10% for sexual TDV, with rates as high as 60% in some investigations.^1,2,3^ Physical TDV is defined as acts of physical aggression toward the dating partner (eg, slapping, hitting, punching), while sexual TDV includes forcing or attempting to force a partner to take part in a sexual act or touching without the partner’s consent.^4^ Research has documented significant associations between sexual and physical TDV and short- and long-term negative outcomes, including depression, suicidality, antisocial behaviors, substance use, injuries requiring medical attention, adult intimate partner violence, and death.^5,6,7,8,9,10^

Given the significant implications for current and long-term effects, physical and sexual TDV constitutes a significant public health problem, and attention to the prevention of TDV is critical.^11^ This issue has gained significant attention in recent years, and an increasing number of programs aimed at reducing sexual and physical TDV has been implemented throughout the last decade.^12^ However, there is currently a lack of a comprehensive and methodologically rigorous meta-analysis of randomized clinical trials (RCT) providing evidence on the capacity of programs to significantly affect physical and sexual TDV. Furthermore, there is a need to identify the target population and intervention factors associated with positive outcomes. It is unclear whether interventions are differentially effective for younger vs older adolescents, based on setting (eg, school-based or community) or whether delivered universally rather than to a selective at-risk group. Important gaps in the literature remain related to the understanding of intervention characteristics important for efficacious interventions (eg, length of interventions, number of sessions, the role of parent involvement) as well as whether the length of time to postintervention assessment is associated with intervention effect size. Such estimates would inform public health decision-making on effective strategies to reduce TDV. The aim of the present study was to conduct a systematic review and meta-analysis of RCTs to evaluate the efficacy of interventions in reducing physical and sexual violence in adolescents’ dating relationships.

## Methods

### Data Sources and Search Strategy

This study was conducted according to the Preferred Reporting Items for Systematic Reviews and Meta-analyses (PRISMA) reporting guideline.^13^ We searched PsycINFO/Eric/PsycArticles, PubMed, and Web of Science databases from inception through April 2021. We combined search terms pertaining to dating violence with those of adolescents and RCTs (eMethods in the Supplement). We also checked the reference lists of earlier systematic reviews and meta-analyses.^14,15,16,17^ Studies were selected independently by 2 reviewers (A.P. and other), and disagreements were resolved by discussion with a third reviewer (H.M.F.). Two authors (H.M.F. and A.P.) also independently searched for protocols in the ClinicalTrials.gov database. After elimination of the duplicates, titles and relevant abstracts were reviewed. The full texts of the remaining records were retrieved to determine whether they met all inclusion criteria.

### Inclusion and Exclusion Criteria

Included studies had a randomized design of any type examining the efficacy of an intervention to reduce TDV compared with a control group. Additionally, at least 1 measure of sexual or physical dating violence perpetration or survivorship needed to be included in the study. Participants had to be 18 years or younger. Nonrandomized comparative studies, observational studies, quasi-experimental studies, and studies published in languages other than English were excluded. We also excluded studies or group comparisons that (1) evaluated nondating violence prevention programs, including those that focused solely on the legislation of sexual-related crimes, and (2) did not follow up the same cohort of participants.

### Data Extraction and Quality Assessment

Two reviewers (A.P. and other) extracted data independently using a standardized study form, which included (1) study information, including geographic location, sample size, percentage of female individuals, and setting; (2) characteristics of participants, including population type, mean age, and history of violence; (3) intervention characteristics, including number of sessions, length, experimental condition, comparison group, and length of follow-up; and (4) outcomes, including measures of physical and sexual dating violence and data for calculating effect sizes. The risk of bias of each included study was assessed separately by 2 of us (A.P. and H.M.F.) with the revised Cochrane collaboration’s tools for RCTs.^18^

### Meta-analysis

We performed pairwise meta-analyses on 3 different outcomes: (1) sexual dating violence; (2) physical dating violence; and (3) a composite measure of sexual and physical dating violence. For each outcome, separate analyses were conducted for survivorship and perpetration. We also combined the measures of physical/sexual violence and perpetration/survivorship into a single composite overall outcome including all studies.

The between-group effect size was computed as the difference between the intervention and control group at posttest or follow-ups up to 3 years by calculating the odds ratio (OR). We chose the OR to minimize the number of conversions to a common effect size because the majority of the included studies reported dichotomous data.

If a trial reported data on multiple follow-ups, the mean effect size was calculated using the appropriate method for multiple outcomes within a study indicated by Borenstein and colleagues^19^ so that each study reported just 1 effect size for each category of outcome analyzed. This method accounts for the correlation between different data gathered on the same individuals, as such observations are not independent from each other.^19^ The same procedure was applied when combining different measures, such as physical and sexual violence or perpetration and survivorship. For multi-arms studies, we applied approximate adjustment methods to reduce unit-of-analysis errors.^20^

If a study did not report the OR, we used available data to calculate the effect size. Effect sizes for dichotomous outcomes were computed, according to the intention-to-treat principle, by reporting the observed number of participants with an event of sexual or physical violence relative to the total number of individuals randomized to that group. Means and standard deviations were used to compute effect sizes for continuous data, which were subsequently converted to ORs.^19^ If only other types of data were available (eg, standardized or unstandardized regression coefficient), we used R version 4.0.3 (R Foundation) to compute the OR. Authors were contacted if a study did not include sufficient data for an effect size calculation, and the study was excluded if they failed to provide such data.

Effect sizes were pooled using random-effects models with a generic invariance method to incorporate the heterogeneity of the differences across the studies. Between-study heterogeneity was measured using the *I^2^* statistics, with values less than 25% indicating low; 25% to 75%, moderate; and more than 75%, considerable heterogeneity.^21^

To evaluate characteristics of interventions associated with positive outcomes, we conducted exploratory subgroup and meta-regression analyses. Subgroup analysis was conducted using a mixed-effects model on categorical moderators that included setting, history of violence, active parental involvement in the intervention, comparison condition, and age. Meta-regression was applied to continuous variables, which included intervention duration, intensity, risk of bias, and length of follow-up.

Sensitivity analyses were performed by serially excluding each study to determine the implications of each individual study for the pooled effect size. Publication bias was assessed through funnel plot^22^ and testing for asymmetry using the Egger test statistic.^23^ The Duval and Tweedy^24^ trim-and-fill procedure was also performed. All analyses were conducted using the Comprehensive MetaAnalysis software version 3.3.070 (Biostat). Statistical tests were 2-sided for the ORs and 1-sided for the Egger test and used a significance threshold of *P *<* *.05.

## Results

### Selection and Characteristics of Included Studies

We screened 975 abstracts, removed 897 (158 duplicates, 739 not relevant), and subsequently retrieved 78 full-text articles. Of these, 19 studies met the inclusion criteria, and 18 studies^25,26,27,28,29,30,31,32,33,34,35,36,37,38,39,40,41,42^ had enough data for calculating effect sizes (eFigure 1 in the Supplement). For the missing trial,^43^ authors were contacted but the requested data were not obtained.

The 18 studies involved a total of 22 781 adolescents. Characteristics of the included RCTs are presented in Table 1. One study was conducted in Europe,^38^ and the remaining were conducted in North America. The mean age of participants ranged from 12.2 to 17.6 years. Thirteen interventions were implemented in schools,^25,27,28,30,31,32,34,35,36,38,39,40,42^ and 5 were implemented in other settings.^26,29,33,37,41^

**Table 1.** Selected Characteristics of Included Studies

Source	Setting	Mean age, y	No. of participants randomized	Female individuals, %	Control group	Duration of intervention	Follow-up (postintervention)^a^
Foshee et al,^25^ 2005	School	13.8	2344	51	Alternative intervention	5 mo	1, 12, 24 mo
Foshee et al,^26^ 2012	Home	NA	464	58	No intervention	3 mo	3 mo
Gonzalez-Guarda et al,^27^ 2015	School	14.3	82	56	Waiting list	3 mo	1 wk, 3 mo, 12 mo
Joppa et al,^28^ 2016	School	15.8	433	54	Waiting list	1 wk	3 mo
Langhinrichsen-Rohling and Turner,^29^ 2012	Health department	17.5	72	100	Waiting list	1 mo	Postintervention
Levesque et al,^30^ 2016	School	NA	3997	53.4	Alternative intervention	2 mo	6 and 12 mo
Miller et al,^31^ 2012	School	NA	2006	0	No intervention	2.5 mo	3 mo
Miller et al,^32^ 2015	School	NA	1012	76.3	No intervention	1 d	3 mo
Miller et al,^33^ 2020	Community	15.5	866	0	Alternative intervention	3 wk	9 mo
Peskin et al,^34^ 2014	School	13	1445	57.8	No intervention	24 mo	Postintervention
Peskin et al,^35^ 2019	School	12.2	1760	52.5	No intervention	3 mo	12 mo
Rizzo et al,^36^ 2018	School	15.7	109	100	Alternative intervention	3 mo	9 mo
Rothman et al,^37^ 2020	Pediatric department	17.6	220	85.5	Alternative intervention	1 d	3 and 6 mo
Sánchez-Jiménez et al,^38^ 2018	School	14.7	1764	47.7	Waiting list	2 mo	6 mo
Taylor et al,^39^ 2010	School	NA	1639	52	No intervention	1 mo	Postintervention and 6 mo
Taylor et al,^40^ 2013	School	NA	2655	53	No intervention	2.5 mo	Postintervention and 6 mo
Wolfe et al,^41^ 2003	Child protection service	15.1	191	49.5	No intervention	4 mo	16 mo
Wolfe et al,^42^ 2009	School	NA	1722	52.8	Alternative intervention	12 mo	30 mo

Abbreviation: NA, not available.

^a^ Follow-up that was analyzed in this meta-analysis.

Primary components of interventions included group discussions/sessions,^29,31,33,36,41^ individual interviews,^32,37^ parent-child activities,^26^ classroom activities,^25,28,38,39,40,42^ and a combination of classroom/group activities and parent-child activities.^27,30,34,35^ The number of sessions ranged from 1 to 24, and the overall length of the intervention ranged from 1 day to 2 years.

Concerning the quality assessment (eFigure 2 in the Supplement), 1 study was estimated to have high risk of bias arising from the randomization process and 2 due to deviations from intended intervention. Eight studies were evaluated as having a high risk of bias in dealing with missing data and 4 in the measurement of the outcome. Five studies prespecified the analysis plan in a registered protocol; thus, selection of the reported result could not be excluded in the remaining studies. The pooled effect sizes for the different outcomes are presented in Table 2.

**Table 2.** Meta-analyses of the Efficacy of Prevention Programs for Physical and Sexual Teen Dating Violence

Variable	No. of trials	Odds ratio (95% CI)^a^	*I*^2^, %	*P* value
Composite overall effect size	18	0.78 (0.69-0.89)	29.9	<.001
Physical perpetration	13	0.74 (0.59-0.92)	65.7	.01
Physical violence survivorship	10	0.78 (0.64-0.95)	64.1	.01
Sexual perpetration	6	0.88 (0.76-1.02)	0.7	.09
Sexual violence survivorship	4	0.88 (0.71-1.08)	45.3	.22
Physical/sexual perpetration	16	0.78 (0.66-0.93)	45.0	.004
Physical/sexual violence survivorship	13	0.77 (0.67-0.89)	48.1	<.001

^a^ According to the random-effects model.

#### Composite Overall Effect Size

The Figure shows the forest plot for the analysis including all 18 studies, whose measures were combined into a single overall composite score of sexual/physical violence survivorship/perpetration (composite overall effect size). The pooled OR for the 18 studies was 0.78 (95% CI, 0.69-0.89; *P* < .001), with low/moderate heterogeneity (*I^2^* = 29.9%). A sensitivity analysis was performed to examine the contribution of each study to the overall effect size, and none of them appeared to markedly influence the observed effect size.

**Figure.**
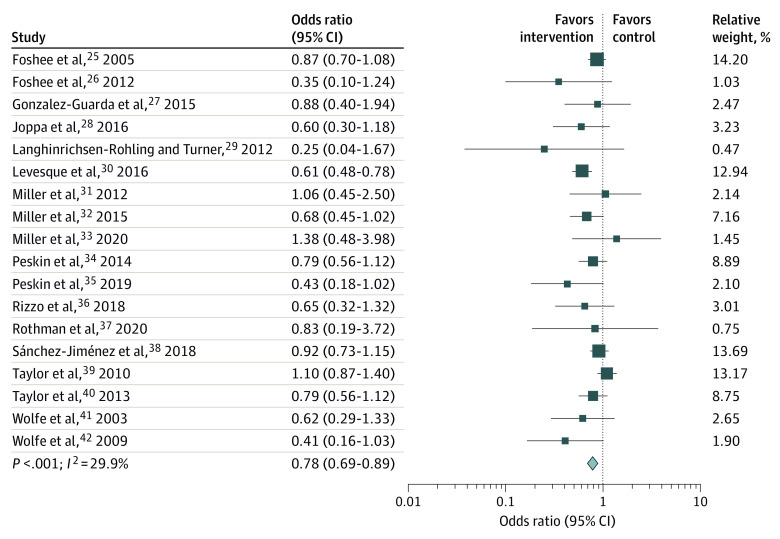
Effect Sizes for Physical and Sexual Dating Violence (Composite Overall Effect)

#### Physical TDV

Thirteen studies^25,26,28,29,30,31,34,35,37,38,39,41,42^ provided a separate measure for physical dating violence perpetration. The pooled OR was 0.74 (95% CI, 0.59-0.92; *P* = .01) in favor of the intervention group compared with the control condition, with moderate heterogeneity (*I^2^* = 65.7%). Concerning physical violence survivorship, the pooled OR for 10 studies^25,26,28,29,30,34,35,38,39,41^ was 0.78 (95% CI, 0.64-0.95; *P* = .01), with moderate heterogeneity (*I^2^* = 64.1%).

#### Sexual TDV

Six studies^25,31,35,37,39,40^ provided a separate measure for sexual dating violence perpetration. Sexual violence perpetration was lower in the intervention group (OR, 0.88; 95% CI, 0.76-1.02) compared with the control condition, but this effect size was not significant. Heterogeneity was low (*I^2^* = 0.7%). Similarly, for sexual violence survivorship, the pooled OR of the 4 studies^25,35,39,40^ favored the intervention condition (OR, 0.88; 95% CI, 0.71-1.08), but it was not significant. Heterogeneity was moderate (*I^2^* = 45.3%).

#### Composite Measures of Physical/Sexual Violence

Sixteen studies^25,26,27,28,29,30,31,33,34,35,37,38,39,40,41,42^ provided a measure of sexual and/or physical perpetration and were combined in a composite perpetration measure. The pooled OR was 0.78 (95% CI, 0.66-0.93; *P* = .004) in favor of the intervention condition with moderate heterogeneity (*I^2^* = 45%).

Concerning the composite survivorship measure, 13 studies^25,26,27,28,29,30,32,34,35,38,39,40,41^ provided a measure of sexual and/or physical survivorship. The pooled OR was 0.77 (95% CI, 0.67-0.89; *P* < .001). Heterogeneity was moderate (*I^2^* = 48.1%).

### Subgroup and Meta-Regression Analyses

We focused the exploratory subgroup (Table 3) and meta-regression (Table 4) analyses on the composite overall effect size including all 18 studies. Trials targeting high-risk adolescents (history of violence) tended to report significantly larger effect sizes compared with universal programs (*Q* = 6.3; *P* = .01). Similarly, interventions that included an active involvement of parents reported significantly larger effect sizes than interventions delivered to adolescents only (*Q* = 5.9; *P* = .01). Significant differences were also observed between interventions delivered to adolescents younger than 15 years compared with those including older participants, with the latter group reporting larger effect sizes (*Q* = 5.2; *P* = .02). There was no difference between trials comparing the experimental intervention with an active and waiting list/no intervention control condition, and between studies conducted in school and different settings. Meta-regression analyses did not show any significant associations between pooled effect sizes and length of intervention, number of sessions, or number of high risk of bias. Conversely, length of follow-up was negatively and significantly associated with effect sizes (coefficient = −0.03; 95% CI, −0.06 to 0; *P* = .03).

**Table 3.** Subgroup Analyses

Variable		No. of trials^a^	Odds ratio (95% CI)^b^		*I*^2^, %	*Q *value	*P* value
Parent involvement in the intervention							
Yes		5	0.65 (0.54-0.79)		0	NA	<.001
No		13	0.86 (0.76-0.98)		0.3	NA	.02
Total between		NA	NA		NA	5.9	.01
Age							
<15 y^c^		9	0.85 (0.73-0.99)		31	NA	.04
>15 y^c^		9	0.65 (0.55-0.78)		0	NA	<.001
Total between		NA	NA		NA	5.2	.02
Prevention program							
Selective program (high-risk adolescents)		5	0.61 (0.49-0.76)		0	NA	<.001
Universal program		13	0.84 (0.74-0.96)		20.7	NA	.01
Total between		NA	NA		NA	6.3	.01
Settings							
School settings		13	0.79 (0.69-0.91)		38.9	NA	.001
Other settings		5	0.66 (0.40-1.09)		0	NA	.10
Total between		NA	NA		NA	0.5	.49
Control condition							
Active control intervention		6	0.72 (0.57-0.92)		34.6	NA	.009
No intervention/waiting list control condition		12	0.82 (0.71-0.96)		22.5	NA	.01
Total between	NA			NA	NA	0.8	.38

Abbreviation: NA, not applicable.

^a^ Analyses on the composite overall association.

^b^ According to the mixed-effects model.

^c^ If a study did not report exact mean age, the measure was approximated from available data.

**Table 4.** Meta-Regression Analyses

Variable	Coefficient (95% CI)^a^	*P* value
Length of follow-up	−0.03 (−0.06 to 0)	.03
Length of intervention	−0.01 (−0.02 to 0)	.24
No. of high risk of bias	−0.14 (−0.35 to 0.06)	.18
No. of sessions	0.03 (−0.02 to 0.08)	.28

^a^ According to the random-effects model. Analyses on the composite overall effect size.

### Publication Bias

Publication bias was observed for all outcomes, including the composite overall effect size (eFigure 3 in the Supplement). Egger test was significant for 3 outcomes: sexual violence survivorship, physical/sexual violence survivorship, and the composite overall effect size. Missing studies identified through the trim-and-fill procedure ranged from 1 to 4 for the different outcomes, but the adjusted ORs remained significant (eTable in the Supplement).

## Discussion

Findings from this systematic review and meta-analysis of RCTs indicate that the implementation of interventions targeting dating violence among adolescents was associated with a significant overall reduction of physical and sexual violence. The magnitude of the effect size can be considered small.^44^ Further analyses showed that the reduction of dating violence was significant for physical perpetration and survivorship. On the contrary, the effect size for sexual violence perpetration and survivorship outcomes failed to reach a significant level. These results might indicate that sexually violent behaviors in dating relationships are more complex and difficult to reduce by prevention programs compared with physical dating violence.^43^ This is also consistent with a previous review,^45^ which did not find evidence to support the efficacy of programs for sexual violence perpetration in adults and adolescents.

Exploratory subgroup analyses of trials stratified by different study-level characteristics identified involvement of parents in the intervention, history of violence, and age of participants as possible sources of heterogeneity in the observed effect sizes. We found that trials that actively engaged parents in the intervention reported significantly greater reduction of dating violence among adolescents compared with studies that delivered the intervention to adolescents only. This is consistent with studies that found family-based programs to be effective in preventing teen health risk behaviors.^46,47^ Furthermore, positive parenting and parental involvement have been found to be associated with lower levels of dating violence among adolescents.^48,49^ Parents do not typically discuss dating violence with their adolescent children,^50^ and our findings suggest that increasing this type of communication by actively involving parents in the intervention could enhance programs’ preventing effects.

This study also showed that effect sizes were significantly larger for trials delivered to high-risk youth than effect sizes for universally delivered interventions. Universally delivered interventions have been shown to be effective in reducing violence in adolescents.^51,52^ It has also been proposed that targeting at-risk youth might result in greater efficacy of interventions owing to the higher baseline levels of hazardous behaviors,^53^ which could potentially explain our findings. However, Foshee and colleagues^43^ found that at-risk youth did not report higher baseline levels of current dating violence and yet experienced a greater reduction. One additional hypothesis is that youth with a history of violence are more receptive to interventions because this is more relevant and salient to their experiences.^34,43^

Furthermore, we found that the age of participants moderated interventions’ effect sizes. Several authors have underscored the importance of intervening at an early age in adolescence to prevent later violent behaviors and support healthy dating relationships.^42,54,55,56^ Our exploratory analysis suggested that early interventions are effective, but the most robust effects might be obtained when targeting older adolescents. Specifically, we found that trials conducted with youth older than 15 years reported a significantly greater reduction of physical and sexual dating violence than studies targeting younger adolescents. This finding could also be explained by the higher levels of dating violence in older adolescents, as longitudinal studies have shown that TDV tends to emerge at an early age (age 13-15 years) and increases as a pattern into late adolescence (age 16-17 years).^42,54,55^

We also found that intervention length and number of sessions were not significantly associated with the efficacy of prevention interventions, similar to other studies on peer violence.^52^ Since the majority of the interventions included in this meta-analysis lasted only a few months, this study indicates that short to medium interventions may significantly reduce sexual and physical violence in teen dating relationships. Nonetheless, our analysis also indicates that an intervention’s effects might slightly decrease over time.

An evaluation of iatrogenic outcomes is essential to provide recommendations for the most appropriate prevention strategies for TDV.^57^ Among the trials included in this meta-analysis, 3 of 19 studies reported iatrogenic effects at follow-ups. Specifically, 1 reported increased physical and sexual violence perpetration with a dating partner^39^; 1 described an increase in individuals who experienced sexual harassment^40^; and 1 reported an increase in individuals who experienced physical and sexual violence among the female subgroup.^27^ Researchers should consider appropriate steps to prevent iatrogenic effects when implementing interventions targeting TDV.

### Limitations

Evidence from this meta-analysis should be interpreted with caution because of some limitations. First, some outcomes categories, such as sexual violence or subgroups, included data from a small number of trials, rendering resultant effect sizes potentially uncertain. Second, we could not include in the meta-analysis 1 study for which we could not compute the effect size. Third, the majority of the studies compared the intervention with waiting list or no intervention condition, and we cannot rule out the possibility that participants were influenced by nonexperimental factors such as greater awareness of the target behaviors. Fourth, we did not analyze other aspects of TDV, such as psychological violence because this dimension and related scales need a critical and systematic evaluation.^58^ Fifth, some of the included studies were potentially at uncertain risk of bias concerning dealing with missing data and selection of the reported results.

## Conclusions

Available data indicate that prevention programs may be effective in reducing physical dating violence among adolescents, but there is unclear evidence of the effect on sexual violence outcomes. Given the low number of studies on sexual violence, further high-quality research is needed. This meta-analysis also identifies different strategies that public health officers and researchers might use to enhance programs’ efficacy, such as tailoring interventions to high-risk and older youth. This could be important from a cost-effectiveness perspective in low-resource settings. Similarly, despite the high variability in programs’ characteristics, our findings suggest that integrating a parental component might improve the efficacy of interventions. Nonetheless, there is need for better designed trials assessing the active ingredients of interventions and the differential efficacy of intervention components. Trials should also test the efficacy of interventions in vulnerable populations such as lesbian, gay, bisexual, transgender, and queer youth, low-income families, and adolescents with disabilities. These studies could support further refinement of effective programs aimed at reducing physical and sexual dating violence among adolescents.

## Notes

Supplement. eMethods. eFigure 1. Flow Diagram Summarizing the Article Selection Process eFigure 2. Estimated Risk of Bias Across All Included Studies eFigure 3. Funnel Plot of Standard Error by Log Odds Ratio (Composite Overall effect) eTable. Publication Bias
