# Supplementary material for: Efficacy of Interventions to Prevent Physical and Sexual Dating Violence Among Adolescents: A Systematic Review and Meta-analysis
Source: JAMA Pediatr. 2021 Nov 29;176(2):1–8. doi: 10.1001/jamapediatrics.2021.4829 (PMC8630665; doi:10.1001/jamapediatrics.2021.4829)
Supplement: Supplement. — eMethods. eFigure 1. Flow Diagram Summarizing the Article Selection Process eFigure 2. Estimated Risk of Bias Across All Included Studies eFigure 3. Funnel Plot of Standard Error by Log Odds Ratio (Composite Overall effect) eTable. Publication Bias [file jamapediatr-e214829-s001.pdf]

Supplemental Online Content

Piolanti A, Foran HM. Efficacy of interventions to prevent physical and sexual dating violence among adolescents: a systematic review and meta-analysis. *JAMA Pediatr*. Published online November 29, 2021. doi:10.1001/jamapediatrics.2021.4829

eMethods.

eFigure 1. Flow Diagram Summarizing the Article Selection Process

eFigure 2. Estimated Risk of Bias Across All Included Studies

eFigure 3. Funnel Plot of Standard Error by Log Odds Ratio (Composite Overall effect)

eTable. Publication Bias

This supplemental material has been provided by the authors to give readers additional information about their work.

© 2021 Piolanti et al. *JAMA Pediatrics.*

eMethods

Search Strategy	
PubMed	("intimate partner violence"[Mesh]) OR (("courtship"[Mesh] OR dating) AND ("Violence"[Mesh] OR "spouse abuse"[Mesh] OR “coercion”[Mesh])) OR ("dating violence") OR ((dating) AND (violence OR abuse OR victimization OR perpetration)) AND ("adolescent"[Mesh] OR "young people" OR student* OR youth or "young adults").<br><br>Filter: Randomized-Controlled Trials
PsycINFO/Eric/PsycArticles	((dating.mp and violence.mp) or (dating.mp and abuse.mp) or dating violence.sh or dating violence.mp or intimate partner violence.sh or intimate partner violence.mp) AND (randomized controlled trials.sh OR randomized.mp or randomized trial.mp or RCT.mp) AND (Adolescent attitudes.sh or adolescent behavior.sh or adolescent.mp or young people.mp OR student* OR youth.sh or youth.mp or young adults.mp).
Web Of Science	(TS=(dating violence OR intimate partner violence OR spouse abuse) OR TS=(dating and violence) OR TS=(dating and abuse) OR TI=(dating and violence) OR AB=(dating and violence)) AND (TS=(randomized Controlled Trials OR randomized OR randomized trial OR RCT) OR TI=(randomized controlled trials OR randomized OR randomized trial OR RCT) OR AB=(randomized controlled trials OR randomized OR randomized trial OR RCT)) AND (TS=(adolescen* or student* or youth or young people or young adults) or TI=(adolescen* or student* or youth or young people or young adults) or AB=(adolescen* or student* or youth or young people or young adults))

© 2021 Piolanti et al. *JAMA Pediatrics.*

eFigure 1. Flow Diagram Summarizing the Article Selection Process

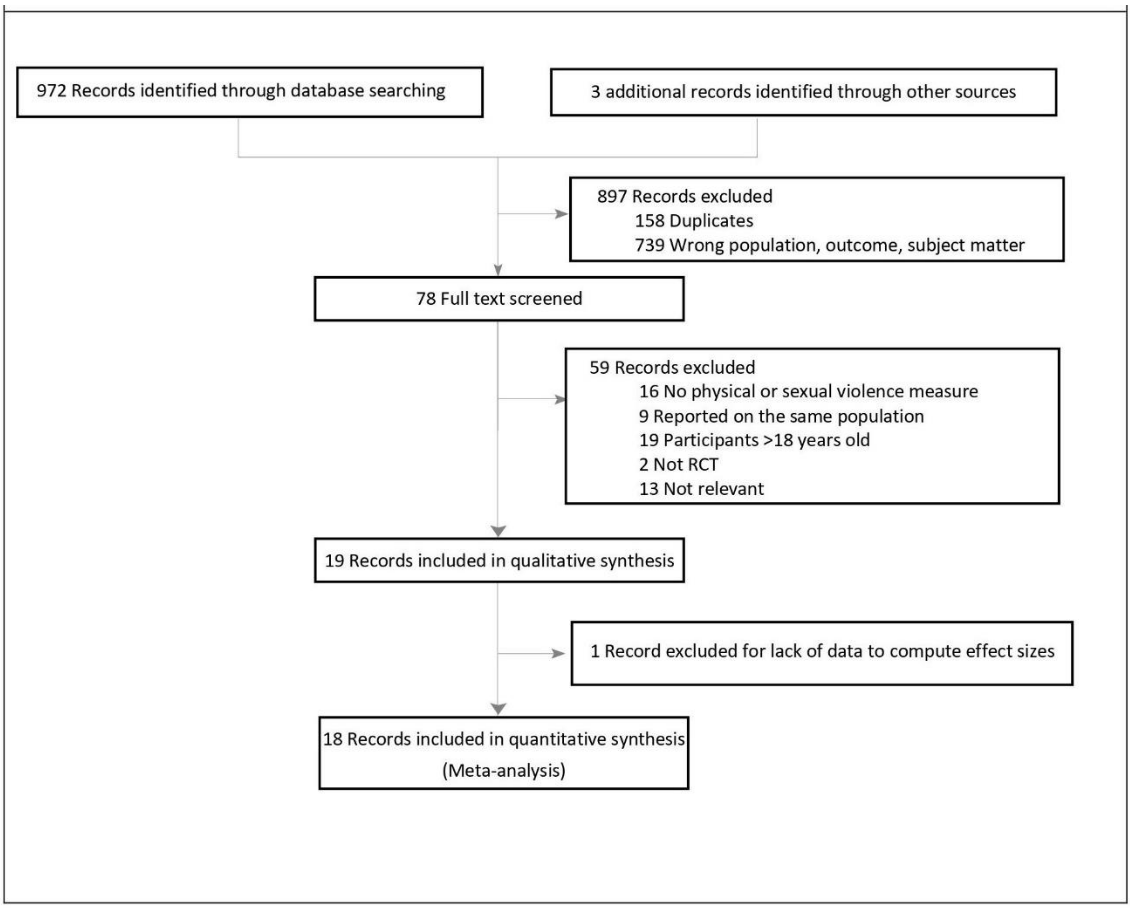

© 2021 Piolanti et al. *JAMA Pediatrics.*

eFigure 2. Estimated Risk of Bias Across All Included Studies

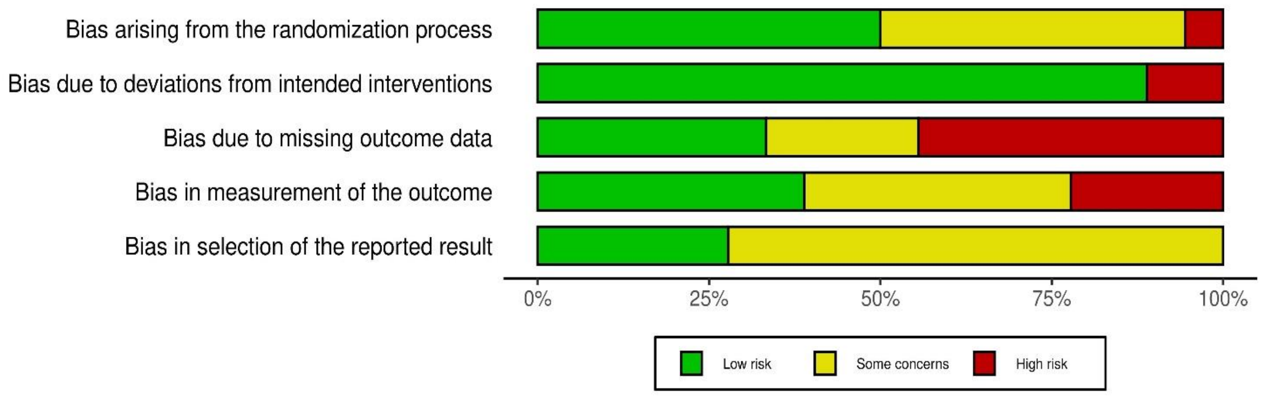

© 2021 Piolanti et al. *JAMA Pediatrics.*

eFigure 3. Funnel Plot of Standard Error by Log Odds Ratio (Composite Overall effect)

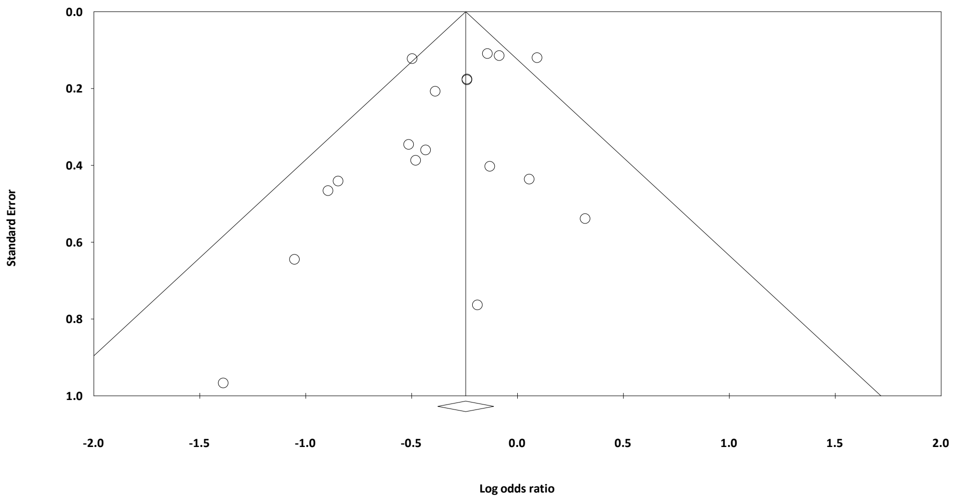

© 2021 Piolanti et al. *JAMA Pediatrics.*

**eTable. Publication Bias**			
Variable	Egger test (*P*)	Trim-and-fill	
		Missing studies (n)	Adjusted Odds Ratio (95% CI)
Composite Overall effect	.04	3	0.80 (0.70 to 0.92)
Physical perpetration	.07	3	0.81 (0.65 to 1.00)
Physical survivorship	.09	3	0.83 (0.68 to 1.00)
Sexual perpetration	.20	2	0.90 (0.78 to 1.04)
Sexual survivorship	.04	1	0.91 (0.70 to 1.17)
Physical/Sexual perpetration	.12	2	0.80 (0.68 to 0.95)
Physical/Sexual survivorship	.04	4	0.81 (0.70 to 0.94)

© 2021 Piolanti et al. *JAMA Pediatrics.*
